# *SMARCC1* Suppresses Tumor Progression by Inhibiting the PI3K/AKT Signaling Pathway in Prostate Cancer

**DOI:** 10.3389/fcell.2021.678967

**Published:** 2021-06-25

**Authors:** Zhao-Ming Xiao, Dao-Jun Lv, Yu-zhong Yu, Chong Wang, Tao Xie, Tao Wang, Xian-Lu Song, Shan-Chao Zhao

**Affiliations:** ^1^Department of Urology, Nanfang Hospital, Southern Medical University, Guangzhou, China; ^2^Guangdong Key Laboratory of Urology, Department of Urology, Minimally Invasive Surgery Center, The First Affiliated Hospital of Guangzhou Medical University, Guangzhou, China; ^3^Department of Radiotherapy, Affiliated Cancer Hospital and Institute of Guangzhou Medical University, Guangzhou, China; ^4^Department of Urology, The Third Affiliated Hospital, Southern Medical University, Guangzhou, China

**Keywords:** *SMARCC1*, prostate cancer, proliferation, epithelial-mesenchymal-transition, *PI3K*/*AKT* pathway

## Abstract

**Background:**

SWI/SNF-related, matrix-associated, actin-dependent regulator of chromatin subfamily C member 1 (*SMARCC1*) protein is a potential tumor suppressor in various cancers. However, its role in prostate cancer (PCa) remains controversial. The aim of this study was to determine the biological function of *SMARCC1* in PCa and explore the underlying regulatory mechanisms.

**Methods:**

The expression of *SMARCC1* was validated in PCa tissues by immunohistochemistry. Meanwhile, function experiments were used to evaluate the regulatory role on cell proliferation and metastasis in PCa cells with *SMARCC1* depletion both *in vitro* and *in vivo*. The expression levels of relevant proteins were detected by Western blotting.

**Results:**

Our finding showed that *SMARCC1* was significantly downregulated in prostate adenocarcinoma, with a higher Gleason score (GS) than that in low GS. The decreased expression of *SMARCC1* was significantly correlated with a higher GS and poor prognosis. Additionally, we found that silencing of *SMARCC1* dramatically accelerated cell proliferation by promoting cell cycle progression and enhancing cell migration by inducing epithelial mesenchymal transition (EMT). Furthermore, depletion of *SMARCC1* facilitated PCa xenograft growth and lung metastasis in murine models. Mechanistically, the loss of *SMARCC1* activated the *PI3K/AKT* pathway in PCa cells.

**Conclusion:**

*SMARCC1* suppresses PCa cell proliferation and metastasis *via* the *PI3K/AKT* signaling pathway and is a novel therapeutic target.

## Introduction

Prostate cancer (PCa) is a common malignancy afflicting elderly males, with over 1.6 million men diagnosed every year (Pernar et al., 2018; Siegel et al., 2021). In America, the morbidity rate of PCa has ranked first in all cancer-related deaths (Siegel et al., 2021). Globally, it is estimated that 366,000 men die of PCa annually, making it the fifth most common cause of cancer-related deaths worldwide (Pernar et al., 2018; Siegel et al., 2021). Multiple mutations in several key epigenetic factors, including Rb1 and BRCA, drive the progression of PCa to aggressive phenotype at terminal stage, characterized by limited survival, revealing an important role of epigenetic dysregulation on PCa progression (Crea et al., 2011; Liu, 2016; Chan et al., 2018; Sheahan and Ellis, 2018; Oh et al., 2019; Wu et al., 2019; Ge et al., 2020; Liang et al., 2020). However, the specific function and profound mechanisms of various epigenetic factors involved in PCa progression are still uncertain. Thus, it is extremely urgent and worthwhile to further explore the role and mechanisms of epigenetic regulators in PCa progression.

The *SWI/SNF*-related, matrix-associated, actin-dependent regulator of chromatin subfamily C member 1 (*SMARCC1*) is one of the core subunits of the SWI/SNF complex involved in epigenetic regulation on genome transcription (Reisman et al., 2009; Shain and Pollack, 2013; Hohmann and Vakoc, 2014; Alver et al., 2017; Lu and Allis, 2017; Savas and Skardasi, 2018). It is regarded as a tumor suppressor in several cancers, including renal, colon, and pancreatic carcinoma (Andersen et al., 2009; Iwagami et al., 2013; Wang et al., 2021). However, its role in PCa remains ambiguous and controversial. Previous studies have shown that *SMARCC1* is upregulated in PCa tissue and may promote the initiation and progression in PCa *via* the transactivating androgen receptor (Hong et al., 2005; Heebøll et al., 2008), whereas a retrospective study on patients with local PCa indicated that *SMARCC1* positive staining in the prostate biopsy samples correlated with prolonged survival, which is indicative of a tumor-suppressive role in PCa (Hansen et al., 2011). These discrepancies may be partly attributed to different cancer models and expression systems.

In this study, we aimed to explore the role of *SMARCC1* in the proliferation and metastasis of PCa by using a series of *in vitro* functional assays and *in vivo* mouse model due to lack of conclusive research on the mechanism of SMRCC1 during PCa progression. Moreover, the potential mechanism by which *SMARCC1* mediates the development and progression of PCa was also investigated.

## Materials and Methods

### Prostate Cancer Tissues

Tumor and matched benign prostatic hyperplasia tissue samples were collected from 100 PCa patients who received radical prostatectomy for prostate cancer at Nanfang Hospital, Southern Medical University (Guangzhou, China). The PCa patients were enrolled in our research project according to the following standards: (1) the patients were diagnosed with PCa before surgery, in accordance with biopsy pathological diagnosis, (2) post-operation pathological examination confirmed the diagnosis of PCa, and (3) the patients were informed and consented to the collection of specimens. Meanwhile, some PCa patients were excluded from our research cohort due to the following criteria: (1) patients with other malignant diseases or a second primary tumor, (2) PCa patients who received preoperative androgen deprivation therapy, chemotherapy, or radiotherapy before surgery, and (3) HIV- or syphilis-positive patients. The relevant clinicopathological data of the patients was also obtained. All patients signed the informed consent, and sample collection was approved by the ethical protocols of the Ethics Committee of Nanfang Hospital, Southern Medical University.

### Immunohistochemistry

The paraffin sections were dewaxed in xylene, rehydrated through an ethanol gradient, and washed in phosphate buffer saline (PBS). After boiling in 10 mM citrate buffer (pH 6) for 10 min for antigen retrieval, the sections were immersed in 3% H_2_O_2_ for 10 min to inhibit endogenous peroxidase. The sections were then blocked with 1% goat serum and incubated overnight with primary antibodies against *SMARCC1* (1:800; Abcam, cat. ab126180, United States), E-Cadherin (1:100; CST, cat. 24E10, United States), *Claudin1* (1:100; CST, cat. 13255T, United States), MMP2 (1:50; HuaBio, cat. ER40806, China), *P504S* (1:100; Proteintech, cat. 15918-1-AP, China), and *Ki67* (1:100; Biossci, cat. BA1063, China) at 4°C. After washing thrice with PBS, the sections were then probed with the secondary antibody for 1 h and rinsed three times with PBS. The DAB Kit (Biossci, cat. BP0770, China) was used for immunostaining, and the nucleus was counterstained with hematoxylin. The sections were then dehydrated through an ethanol gradient and sealed with neutral balsam. The stained sections were observed under a light microscope (BX53, Olympus, Japan) fitted with a digital camera (DP72, Olympus, Japan). The *in situ* *SMARCC1* expression level was analyzed independently by two pathologists blinded to the patients’ clinical information. The staining intensity was scored as negative (0), weak (1), medium (2), or strong (3), and the percentage of positively stained area was scored as 0 (0%), 1 (1–25%), 2 (26–50%), 3 (51–75%), and 4 (76–100%). The total staining score was calculated by multiplying the intensity and positivity scores, and the samples were graded as low (0–5), medium (6–8), and high (9–12) expression accordingly. In addition, low staining intensity in tissue was classified as negative, while both medium and high staining intensity were classified as positive.

### Bioinformatics Analyses

The disease-free survival data was extracted from the Gene Expression Profiling Interactive Analysis (GEPIA) database^1^ and analyzed using R. The cutoff was set as 30% high vs. 70% low.

### Cell Culture

RWPE-1, LNCAP, C4-2, PC3, 22RV1, and DU145 cell lines were obtained from the Cell Bank of Typical Culture Preservation Committee of the Chinese Academy of Sciences (Shanghai, China). The RWPE-1 cells were cultured in primary keratinocyte culture medium (iCell Bioscience Inc., cat. PriMed-iCell-010, China), and the other cell lines were cultured in RPMI 1640 (Gibco, cat. 11875-093, United States) supplemented with 10% fetal bovine serum (FBS; Gibco, cat. 10270-106, United States) and 1% penicillin–streptomycin solution (Gibco, 15140-122, United States) at 37°C under 5% CO_2_. As per experimental requirements, the cells were treated with 20 ng/ml LY294002 (Macklin, cat. HY-10108, China) in dimethyl sulfoxide for 24 h (Bao et al., 2015).

### *SMARCC1* Knockdown

The cells were seeded in six-well plates at a density of 0.5 × 10^6^ cells/well and transfected 24 h later with *SMARCC1* siRNA using Lipofectamine 3000 (Invitrogen, United States). The cells were harvested 48 h after transfection, and *SMARCC1* mRNA and protein levels were analyzed by quantitative real-time polymerase chain reaction (qRT-PCR) and western blotting. The lentivirus vector containing the *SMARCC1* shRNA was synthesized by Vigene Biosciences Inc. (China). Briefly, 0.5 × 10^6^ cells were plated in six-well plates and infected 24 h later with the virus at a multiplicity of infection (MOI) of 30 in serum-free medium. Fresh complete medium containing 2 μg/ml puromycin was added 48 h after transfection, and the cells were cultured for 4 days. The sh-*SMARCC1* cell lines with stable knockdown were detected by qRT-PCR and western blotting. siRNA targeted to *SMARCC1*: 5′-CCUCACAAGACGAUGAAGATT-3′ 5′-UCUUCAUCGUCUUGUGAGGTT-3′ was synthesized in GenePharma Co., Ltd (Suzhou, CHINA) and sh RNA contained in lentivirus vectors was designed according to siRNA sequence.

### RNA Extraction and qRT-PCR

RNA was extracted using Trizol reagent (Invitrogen, cat. 15596018, United States) and reverse-transcribed to cDNA using PrimeScript RT Master Mix Kit (Takara, cat. RR036A, Japan) according to the manufacturer’s instructions. The cDNA was amplified with the PrimeScript^TM^ RT-PCR Kit (Takara, cat. DRR015A, Japan) on Applied Biosystems^TM^ 7500 fast Dx real-time PCR cycler (Thermofisher, United States) according to the manufacturer’s protocol. GAPDH was used as the internal control, and relative expression levels were calculated by the 2^–ΔΔ*CT*^ method. Primer sequence was obtained from website primerbank (https://pga.mgh.harvard.edu/primerbank/) and shown as following: *SMARCC1* (Forward sequence: 5′-AGCTGTTTATCGACGGAAGGA-3′; Reverse sequence: 5′-GCATCCGCATGAACATACTTCTT-3′); GAPDH (Forward sequence: 5′-GGAGCGAGATCCCTCCAAAAT-3′; Reverse sequence: 5′-GGCTGTTGTCATACTTCTCATGG-3′).

### Protein Extraction and Western Blotting

The suitably treated cells were homogenized in radio-immunoprecipitation assay (RIPA) buffer supplemented with phenylmethylsulfonyl fluoride (KGP250, KeyGEN BioTECH, Nanjing, China) at the ratio of 1,000:1, along with protease inhibitor (Mecklin, cat. P885281, China) and phosphatase inhibitor (FDbio, cat. FD7186, China), each at 100:1 ratio. The cells were lysed by ultrasonication for 20 min and centrifuged at 12,000 rpm for 5 min at 4°C. The nuclear and cytoplasmic proteins were fractionated using the nucleus extraction kit (Pythonbio, cat. AAPR285, Guangzhou, China). Briefly, 1 × 10^7^ of cells were lysed using a specific buffer at 4°C for 20 min and centrifuged for 5 min. The cytoplasmic fraction was removed, and the nuclear fraction was washed thrice with PBS. Protein was extracted from both fractions using RIPA buffer as described above. The respective supernatants were aspirated, mixed with × 5 loading buffer (FDbio, cat. FD0006, China) at a ratio of 1:4, and denatured by boiling. An equal amount of protein per sample was separated by 10% sodium dodecyl sulfate polyacrylamide gel electrophoresis and transferred to polyvinylidene fluoride membrane (Millpore, cat. MB0323, United States). After blocking with 10% milk or bovine serum (for phosphorylated proteins), the membranes were incubated overnight with primary antibodies specific for *SMARCC1* (1:500; CST, cat. D7F83, United States), *Cyclin D1* (1:500; CST, cat. 92G2, United States), *Cyclin E1* (1:500; CST, cat. HE12, United States), *CDK6* (1:500; CST, cat. DSC83, United States), *p21* (1:500; CST, cat. 12D1, United States), *p27* (CST, cat. D96C12, United States), *E-Cadherin* (1:500; CST, cat. 24E10/4A2, United States), *N-Cadherin* (1:500; CST, cat. D4R1H, United States), *Vimentin* (1:500; CST, cat. D21H3, United States), *β-catenin* (1:500; CST, cat. D10A8, United States), *Snail* (1:500; CST, cat. C15D3, United States), *Slug* (1:500; CST, cat. C19G7, United States), *Zeb1* (1:500; CST, cat. D80D3, United States), *Zeb2* (1:500; ABclone, cat. A5705, CHINA), *Akt* (1:500; CST, C67E7, United States), *p-Ak^*Ser473*^* (1:500; CST, cat. D9E, United States), *p-Akt^*Thr308*^* (1:500; CST, cat. D25E6, United States), *GAPDH* (1:1,000; Proteintech, cat. 104941-AP, China; CST, cat. D4C6R, United States), and *H3* (1:500; Bioss, cat. bs-0349R, China) at 4°C. Total and cytoplasmatic proteins were normalized to GAPDH, and nuclear protein was normalized to histone *H3*. PVDF membranes were washed with PBS-tween solution (PBST) and incubated with horseradish peroxidase-conjugated secondary antibody for 1 h. After washing thrice with PBS, the positive bands were detected by FDbio-Dura enhanced chemiluminescence kit (FDbio, cat. FD8020, China).

### Cell Viability Assay

The cells were seeded in 96-well plates at a density of 1,000 cells/well in triplicates, and viability was measured using the Cell Counting Kit 8 (Dojindo, cat. CK04, Japan) according to the manufacturer’s instructions. The absorption at 450 nm was detected using Microplate Reader Synergy Neo2 (Biotek, United States).

### Colony Formation Assay

The cells were seeded in six-well plates at a density of 3 × 10^3^ cells/well in complete medium and cultured for 12–14 days till colonies were visible. The colonies were fixed with methanol and stained using the Wright–Giemsa kit (Baso, cat. BA4017, China). Colonies harboring >50 cells were counted under a microscope.

### Flow Cytometry Assay

The cell cycle distribution was analyzed using a specific detection kit (KeyGENE BioTECH, cat. KGA512, Nanjing, China). Briefly, the suitably treated cells were harvested and fixed in 75% ethanol for 48 h. After washing with PBS, the cells were incubated with ribonuclease at 37°C for 30 min to remove intracellular RNA. The DNA was stained with propidium iodide (PI), and cells were acquired in the FACS Calibur flow cytometer (Bioscience, United States) to measure the DNA content.

### EDU Assay

The cells were incubated with EDU solution (EDU assay kit, cat. C10310-1, RiboBio, China) diluted 1:1,000 in complete medium for 2 h. After washing thrice with PBS, the cells were fixed with 4% paraformaldehyde for 10 min and neutralized by 2 mg/ml glycine. The cells were then permeabilized with 0.5% Triton and incubated with the kit reaction agent for 30 min. After washing thrice with 0.5% Triton and once with methanol, the cells were counterstained with Hoechst 33342 (diluted 1:1,000 with ddH_2_O) for 10 min to stain the nucleus and washed thrice with PBS. The EDU-positive cells were counted under an inverted microscope (Olympus, cat. 1X71, Japan) fitted with DP72 camera (Olympus, Japan).

### Immunofluorescence Assay

The cells were seeded in a 24-well plate at a density of 0.2 × 10^6^ cells/well and fixed with 4% paraformaldehyde for 10 min following 24 h of incubation. After neutralizing with 2 mg/ml glycine, the fixed cells were permeabilized with 0.5% Triton for 10 min and washed thrice with PBS. The cells were then blocked with 10% BSA for 1 h and incubated with primary antibodies against E-Cadherin (1:100; CST, cat. 24E10, United States), N-Cadherin (1:100; CST, cat. D4R1H, United States), Vimentin (1:100; CST, cat. D21H3, United States), β-catenin (1:200; CST, cat. D10A8, United States), and p-Akt^*Ser473*^ (1:200; CST, cat. D9E, United States) at 37°C for 2 h. After washing thrice with PBST for 5 min, the cells were incubated with the secondary antibody at 37°C for 1 h. The stained cells were washed thrice with PBST and observed under an inverted microscope (Olympus, cat. 1X71, Japan) fitted with DP72 camera (Olympus, Japan).

### Wound Healing Assay

The cells were seeded in a six-well plate at a density of 0.8 × 10^6^ cells/well and cultured for 48 h till 90% confluent. The monolayer was scratched longitudinally with a 10-μl pipette tip, and the debris was removed by washing thrice with PBS. Fresh serum-free medium was added, and the wound region was photographed at 0, 12, and 24 h after scratching under an inverted microscope (Olympus, cat. 1X71, Japan) with DP72 camera (Olympus, Japan). The extent of wound healing was measured using Image J (NIH, United States).

### Transwell Assay

The cells were seeded in the upper chambers of 24-well Transwell inserts (Corning, cat. 3422, United States) at a density of 1 × 10^5^ cells/well in serum-free medium, and the lower chambers were filled with complete medium supplemented with 20% FBS. After culturing for 48 h, the unmigrated cells on the upper surface of the membranes were swabbed with a cotton ball, and the migrated cells on the lower surface were fixed with methanol for 10 min and stained with Wright–Giemsa kit (Baso, cat. BA4017, China). The number of migrated cells were counted in three random fields under a BX53 microscope (Olympus, Japan) fitted with DP72 camera (Olympus, Japan).

### Mouse Xenograft Models

BALB/c nude mice were obtained from Guangdong Medical Laboratory Animal Center (Guangdong, China). All animal experiments were approved by the Ethical Committee of Southern Medical University and conducted according to the NIH Guide for the Care and Use of Laboratory Animals. The subcutaneous xenograft was established in 4-week-old mice by subcutaneously injecting 5 × 10^6^ cells into their flanks (four mice per group). The animals were sacrificed 33 days after inoculation, and the tumors were weighed, measured, and photographed with SX720 HS camera (Canon, Japan). For the lung metastasis model, 0.2 × 10^6^ cells were injected intravenously into 6-week-old mice through the tail vein. The animals were sacrificed 6 weeks later, and the lung tissues were removed and photographed with SX720 HS camera (Canon, Japan). The tissues were fixed in 10% formalin, embedded in paraffin, and cut into sections. Hematoxylin–eosin staining was performed as per standard protocols and photographed under the BX53 microscope (Olympus, Japan) using DP72 camera (Olympus, Japan). IHC was performed to detect the *in situ* expression of proliferation and metastasis markers as described. The area invaded by the tumor was calculated by Image J software (NIH, United States).

### Statistical Analysis

All statistical analyses were performed using GraphPad 7.0 (GraphPad Software Inc., United States) or SPSS 22.0 (IBM Corp., United States). Chi-square test or Fisher’s exact test was used to determine the correlations between the *in situ* protein abundance and the clinicopathological factors in PCa tissues. Numerical data were expressed as means ± standard error of mean. Differences between variables were confirmed by two-tailed Student’s *t*-test or one-way analysis of variance (ANOVA) for continuous variable groups. When ANOVA was significant, *post-hoc* testing of differences between groups was carried out using the least significant difference test. Survival curves were plotted using Kaplan–Meier’s method and compared by the log-rank test. A *P*-value < 0.05 was considered statistically significant.

## Results

### *SMARCC1* Is Downregulated in PCa Tissues With GS More Than 7 and Correlates With Poor Prognosis

**FIGURE 1:**
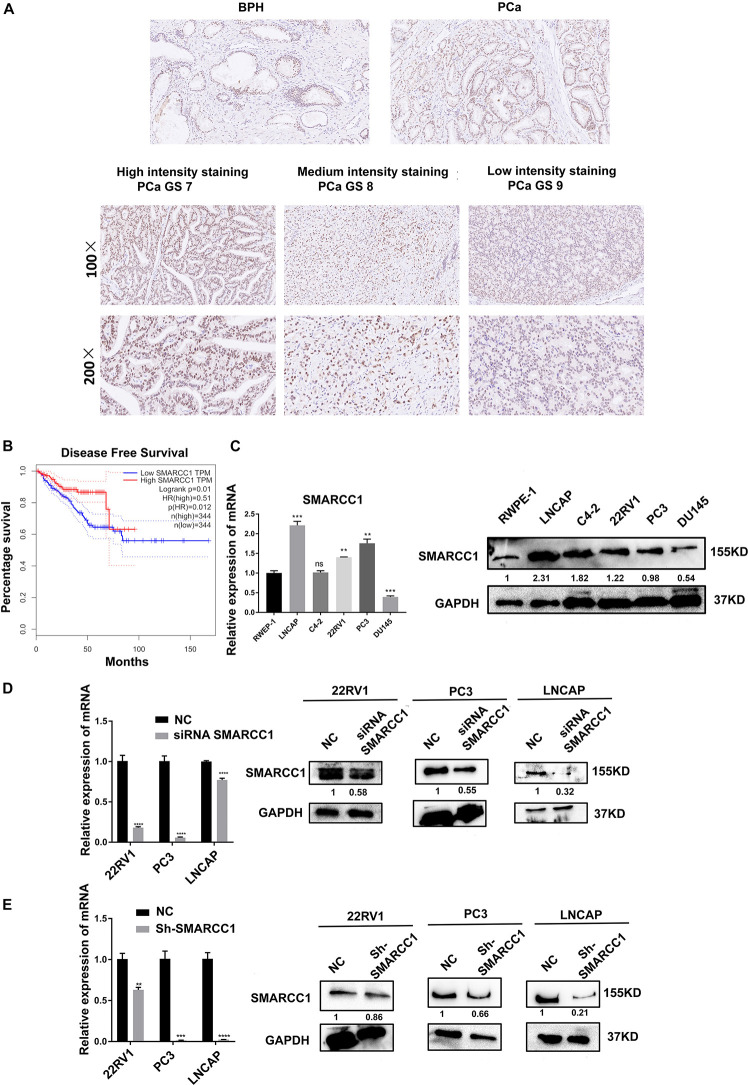
*SMARCC1* is downregulated in PCa tissues and correlates with poor prognosis. **(A)** Representative images of BPH and PCa tissues with Gleason score 7, 8, and 9 showing *in situ SMARCC1* expression (magnification, ×100). **(B)** Disease-free survival of PCa patients with a different *SMARCC1* expression from the GEPIA database. **(C)** Endogenous expression of *SMARCC1* mRNA in immortalized normal prostatic epithelial cells and PCa cell lines. **(D,E)** *SMARCC1* mRNA **(D)** and protein **(E)** levels in PCa cells transduced with *SMARCC1* shRNA lentivirus. Relative mRNA expression levels are shown in the bar graph, and gray density values of proteins are indicated under the corresponding bands. All data are presented as mean ± SEM of at least three independent experiments. ***p < 0.001; ****p < 0.0001.

Compared with that in matched non-tumorous tissues of BPH, *in situ* *SMARCC1* expression in PCa tissue was slightly upregulated without statistical significance (Figure 1A and Table 1). In PCa cases with GS > 7, *in situ* *SMARCC1* expression was significantly downregulated (Figure 1A and Table 2). Consistent with this, the GEPIA data showed that the high expression of *SMARCC1* positively correlates with prolonged disease-free survival in PCa patients (Figure 1B). Thus, the loss of *SMARCC1* portends poor prognosis and disease progression in PCa. Moreover, endogenous expression of *SMARCC1* in PCa cell lines was validated using western blotting assay and was further knocked down using transient transfection of siRNA or lentivirus vector containing short hairpin RNA (Figures 1C–E).

**TABLE 1:** *SMARCC1* expression profile in tissue from BPH and PCa.

Origin type of tissue specimens	Immunohistochemical staining intensity		*χ*^2^	*P*-value
	Negative (n, %)	Positive (n, %)		
BPH	37 (74)	13 (26)	2.487	0.115
PCa	61 (61)	39 (39)		

**TABLE 2:** Correlation between *SMARCC1* expression and pathological characteristics of PCa.

Pathological characteristics	Immunohistochemical staining intensity			*χ*^2^	*P*-value
	Low (n, %)	Medium (n, %)	Strong (n, %)		
**Age**
≤60	4 (100)	–	–	2.664	0.264
>60	57 (59.4)	31 (32.3)	8 (8.3)		
**Gleason score**
≤7	27 (50)	23 (42.6)	4 (7.4)	**7.469**	**0.024**
>7	34 (73.9)	8 (17.4)	4 (8.7)		
**T stage**
T1–2	7 (46.7)	6 (40)	2 (13.3)	1.547	0.461
T3–4	50 (63.3)	23 (29.1)	6 (7.6)		
**Metastasis to lymph nodes**
Yes	17 (73.9)	6 (26.1)	–	3.439	0.179
No	44 (57.1)	25 (32.5)	8 (10.4)		
**Invasion to seminal vesicle**
Yes	37 (58.7)	21 (33.4)	5 (7.9)	0.375	0.829
No	21 (63.6)	9 (27.3)	3 (9.1)		
**AJCC clinicopathological stage**
I–IIc	6 (50)	4 (33.3)	2 (16.7)	3.348	0.501
IIIa–IIIc	49 (60.5)	26 (32.1)	6 (7.4)		
IVa–IVb	6 (85.7)	1 (14.3)	0		
**ERG staining**
Negative	37 (63.8)	15 (25.9)	6 (10.3)	3.477	0.176
Positive	9 (42.9)	10 (47.6)	2 (9.5)		

*Bold values illustrate statistical significance.*

### *SMARCC1* Inhibits PCa Cell Proliferation *in vitro*

**FIGURE 2:**
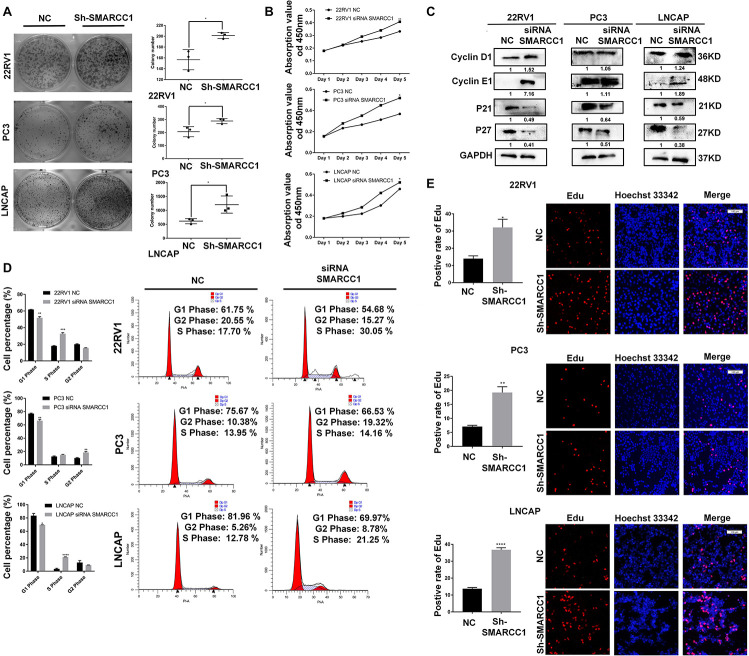
*SMARCC1* knockdown increased PCa cell viability and proliferation. **(A)** Number of colonies formed by control and *SMARCC1* knockdown PCa cells. **(B)** Percentage of viable PCa cells treated as indicated. **(C)** CKI and cyclin mRNA and protein levels in the indicated groups. Relative mRNA expression levels are shown in the bar graph, and gray density values of proteins are indicated under the corresponding bands. **(D)** Bar graph showing the distribution of PCa cell lines in the different cell cycle stages. **(E)** Representative images (right panel, scale bar: 100 μm) of EDU-stained cells and bar graph showing the percentage of EDU-positive cells in the S phase. All data are presented as mean ± SEM of at least three independent experiments. *p < 0.05; **p < 0.01; ***p < 0.001; ****p < 0.0001.

*SMARCC1* silencing significantly increased the viability of PCa cells as well as the number of colonies formed *in vitro* (Figures 2A,B). Loss of *SMARCC1* upregulated *cyclinD1/E1* and downregulated the *cyclin-dependent kinase inhibitors* (*CKIs*) *p21* and *p27*, which indicated accelerated cell cycle transition (Figure 2C). Consistent with this, EDU incorporation and PI staining assays showed that *SMARCC1* knockdown increased the percentage of cells entering S and G2 phases and decreased that of cells remaining in G1 phase (Figures 2D,E). In conclusion, *SMARCC1* loss in PCa cells accelerated cell cycle progression and increased their proliferation by downregulating *CKIs* and activating *cyclin D1/E1*.

### *SMARCC1* Silencing Accelerates the Metastasis of PCa Cells by Inducing EMT

**FIGURE 3:**
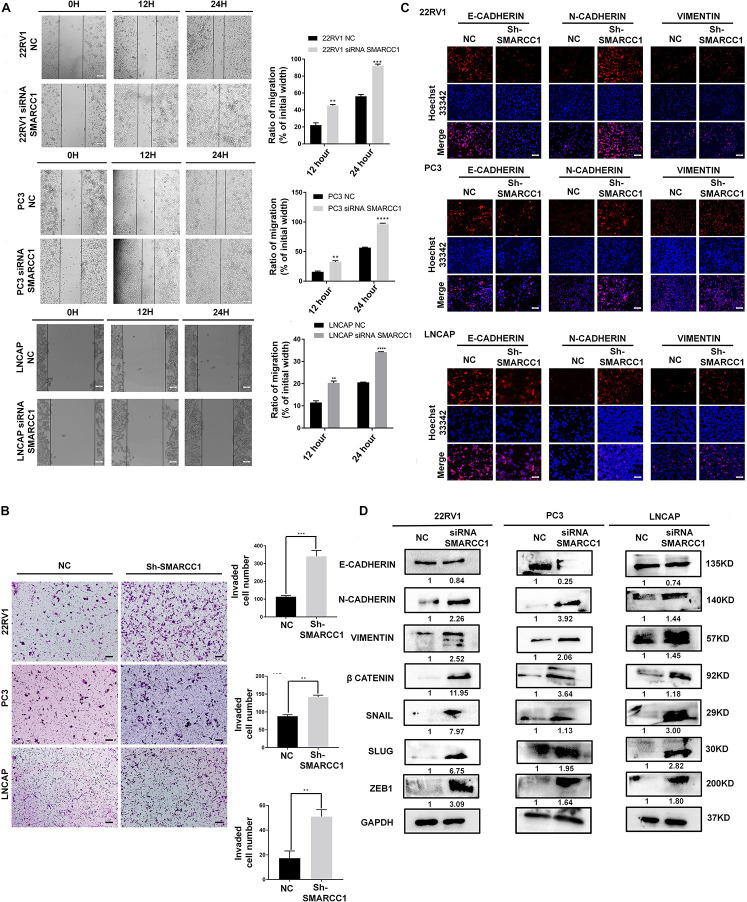
*SMARCC1* knockdown induced EMT, migration and metastasis of PCa. **(A)** Representative images showing *in vitro* wound area coverage by control and *SMARCC1*-knockdown PCa cells (left panel; magnification 100×; Scale bar – 10 μm) and percentage of migration (right panel). **(B)** Representative images showing migration of PCa cells in the Transwell assay (magnification 100×; Scale bar – 50 μm) and percentage of migrating cells. **(C)** Representative immunofluorescence images showing E-cadherin, N-cadherin and vimentin expression in PCa cell lines (Scale bar: 100 μm). **(D)** Expression of EMT markers and transcription factors involved in EMT in the indicated groups. Gray density values of proteins are indicated under the correspondent bands. All data are presented as mean ± SEM of at least three independent experiments. **p < 0.01;***p < 0.001; ****p < 0.0001.

Transwell and wound healing assays showed that loss of *SMARCC1* significantly increased the migration of PCa cells *in vitro* (Figures 3A,B). Consistent with this, *SMARCC1* knockdown markedly increased the expression of mesenchymal markers, including vimentin and N-cadherin, and decreased that of *E-cadherin* (Figures 3C,D). In addition, EMT-related transcription factors, including *Slug*, *Snail*, and *Zeb1*, were also upregulated in the *SMARCC1*-knockdown cells (Figure 3D). In conclusion, *SMARCC1* loss promoted the EMT of PCa cells, thereby inducing migration and metastasis.

### Knockdown of *SMARCC1* Promotes the Growth and Metastasis of Human PCa Cells *in vivo*

**FIGURE 4:**
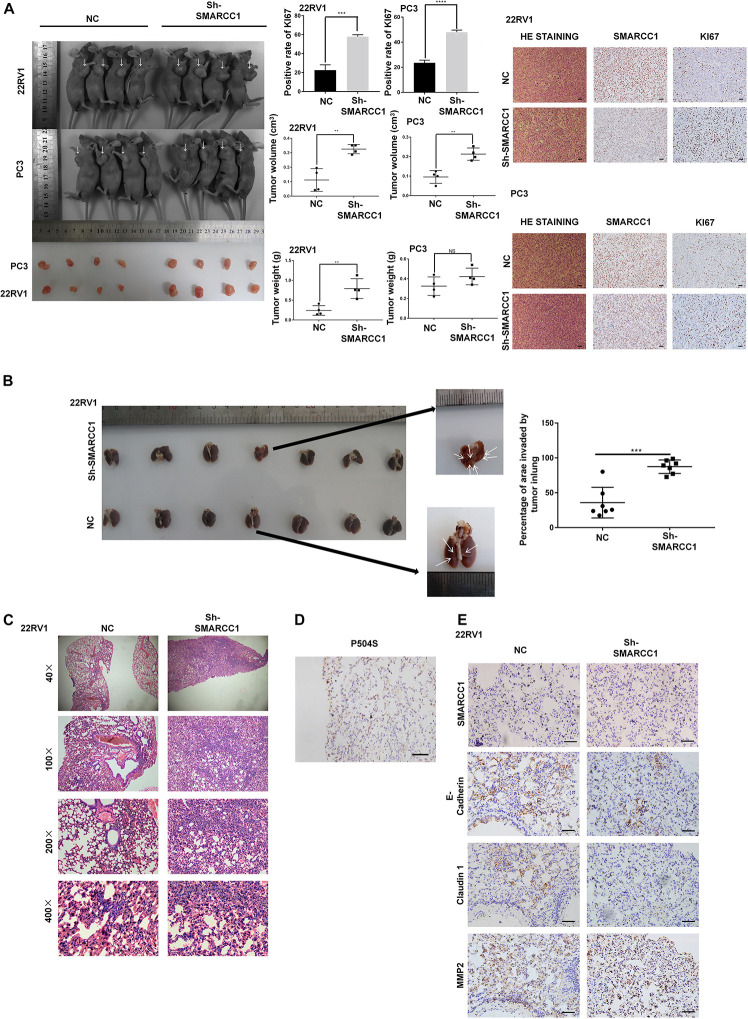
*SMARCC1* knockdown promoted tumor growth and metastasis *in vivo*. **(A)** Left panel: representative images of subcutaneous tumor xenografts in the indicated groups and scatter plots showing tumor volume and weight. Right panel: representative immunohistochemistry (IHC) images showing *SMARCC1* and KI67 expression (magnification, ×200; scale bar, 20 μm) and bar graph showing the percentage of KI67+ cells. **(B,C)** Schematic illustration of lung metastasis model. Representative images of H&E-stained tumor nodules and scatter plots showing the percentage of lung area invaded by PCa cells in the indicated groups. **(D)** Representative IHC images of metastatic nodules showing the positive staining of P504S indicative of prostate origin (magnification, ×400; scale bar, 20 μm). **(E)** Representative IHC images of metastatic nodules showing the *in situ* expression of *SMARCC1*, MMP2, E-cadherin, and claudin1 (magnification, ×400; scale bar, 20 μm). All data were presented as mean ± SEM of at least three independent experiments. **p < 0.01; ***p < 0.001; ****p < 0.0001.

To validate the potential impact of *SMARCC1* depletion on PCa cell proliferation *in vivo*, PC-3/sh-*SMARCC1* cells and PC-3/sh-Ctrl as well as 22Rv1/sh-*SMARCC1* cells and 22Rv1/sh-Ctrl cells were injected subcutaneously in nude mice. Tumors in mice implanted with sh-*SMARCC1* cells grew faster than control cells. *SMARCC1* knockdown cells exhibited significantly larger tumor volume and weight than control cells (Figure 4A). H&E staining showed the histopathological features of the tumor tissues. The positive rate of proliferation mark, Ki-67, was dramatically higher in xenografts with sh-*SMARCC1* cells by IHC staining (Figure 4A). These results provided evidence that *SMARCC1* may be a remarkable determinant for PCa cell growth. As for tumor metastasis *in vivo*, a tail vein xenograft model was generated. The tumor presence was validated by histological examination. The results demonstrated that mice injected with 22Rv1/*SMARCC1* shRNA cells produced more lung colonization compared to those with that of the control cells (Figures 4B,C). As shown in Figure 4D, *P540S* was stained to confirm the neoplasms’ histologic type and origin of lung colonization. Moreover, we also found that the *SMARCC1*-knockdown pulmonary tumor nodules showed significantly higher levels of the matrix *metalloproteinase* (MMP2) and low levels of epithelial markers including *E-Cadherin* and *Claudin 1* (Figure 4E).

### Loss of *SMARCC1* Activates the *PI3K/AKT* Pathway in PCa Cells

**FIGURE 5:**
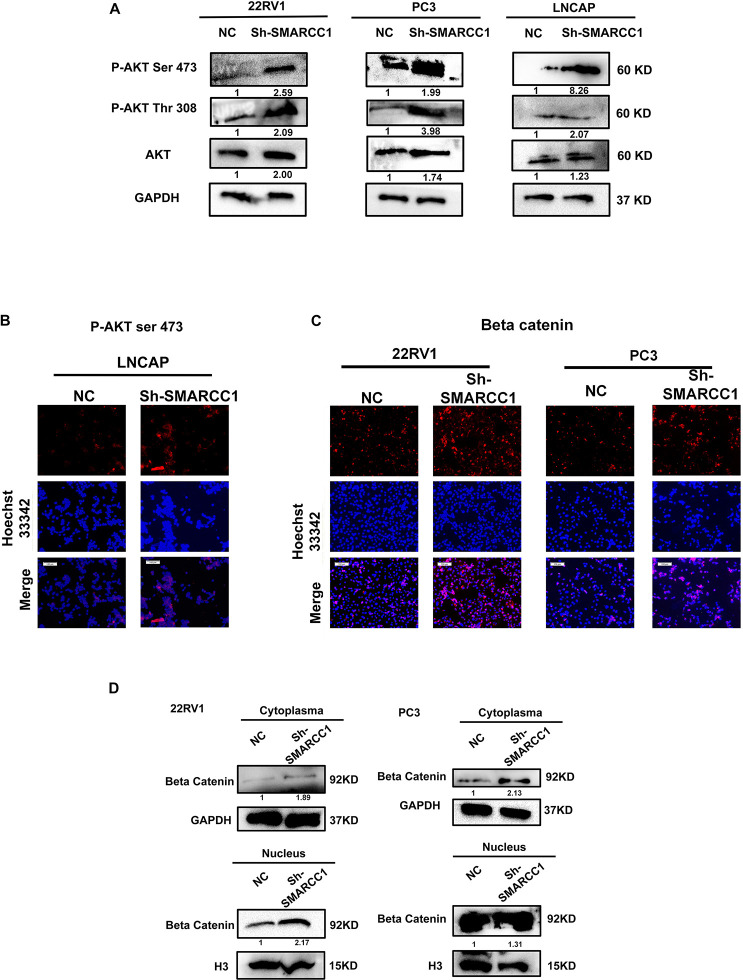
*SMARCC1* knockdown activated the PI3K/AKT pathway in PCa cell lines. **(A)** Immunoblots showing the expression levels of phosphorylated PI3K/AKT mediators. The gray density values are indicated under the corresponding bands. **(B)** Representative immunofluorescence images showing the *in situ* expression of p-AKT^*ser*–473^ in the LNCAP cell line (scale bar, 100 μm). **(C)** Representative immunofluorescence images showing the expression of β-catenin in the cytoplasm and nucleus of 22RV1 and PC3 cell lines (scale bar, 100 μm). **(D)** Immunoblots showing the expression levels of β-catenin in the cytoplasmic and nuclear fractions. The gray density values are indicated under the corresponding bands.

**FIGURE 6:**
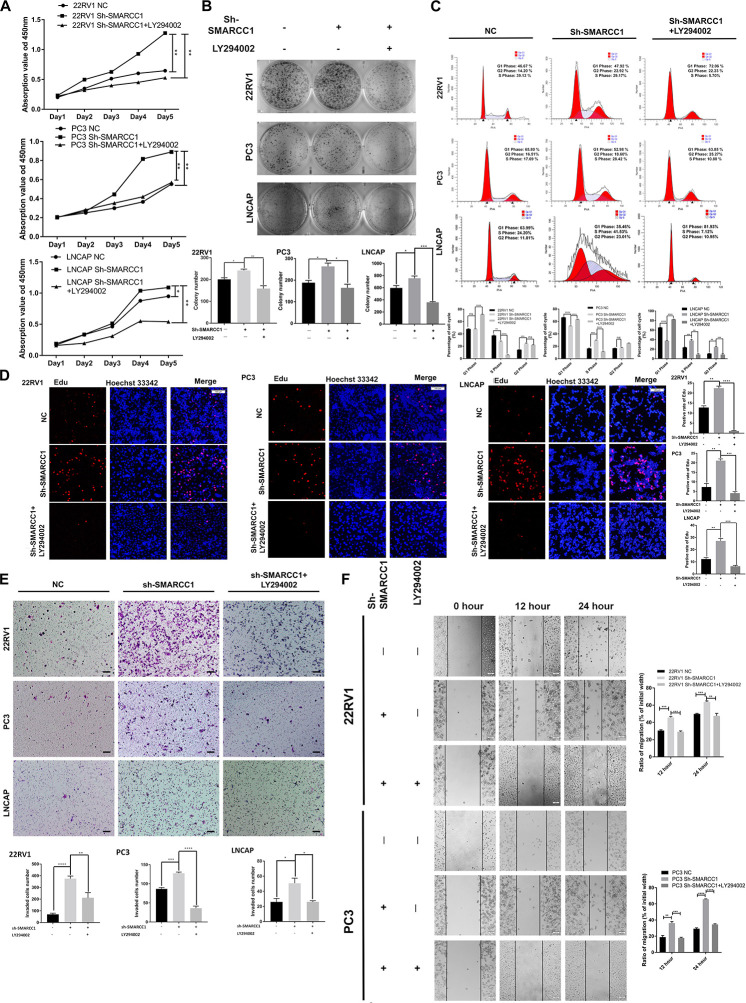
PI3K/AKT pathway blockade reversed the phenotypic effect of *SMARCC1* knockdown. **(A)** Viability rate, **(B)** number of colonies, **(C)** cell cycle distribution, and **(D)** Representative image of EdU for LNCAP with Sh-*SMARCC1* was selected from area presented in Figure 2E for LNCAP cell line with Sh-*SMARCC1*. **(E)** Transwell assay on *SMARCC1* knocked-down PCa cell lines treated with ly294002 (scale bar – 100 μm). **(F)** Wound healing assay on migration ability of *SMARCC1* knocked-down PCa cell lines treated with ly294002. All data are presented as mean ± SEM of at least three independent experiments. *p < 0.05; **p < 0.01; ***p < 0.001; ****p < 0.0001.

**FIGURE 7:**
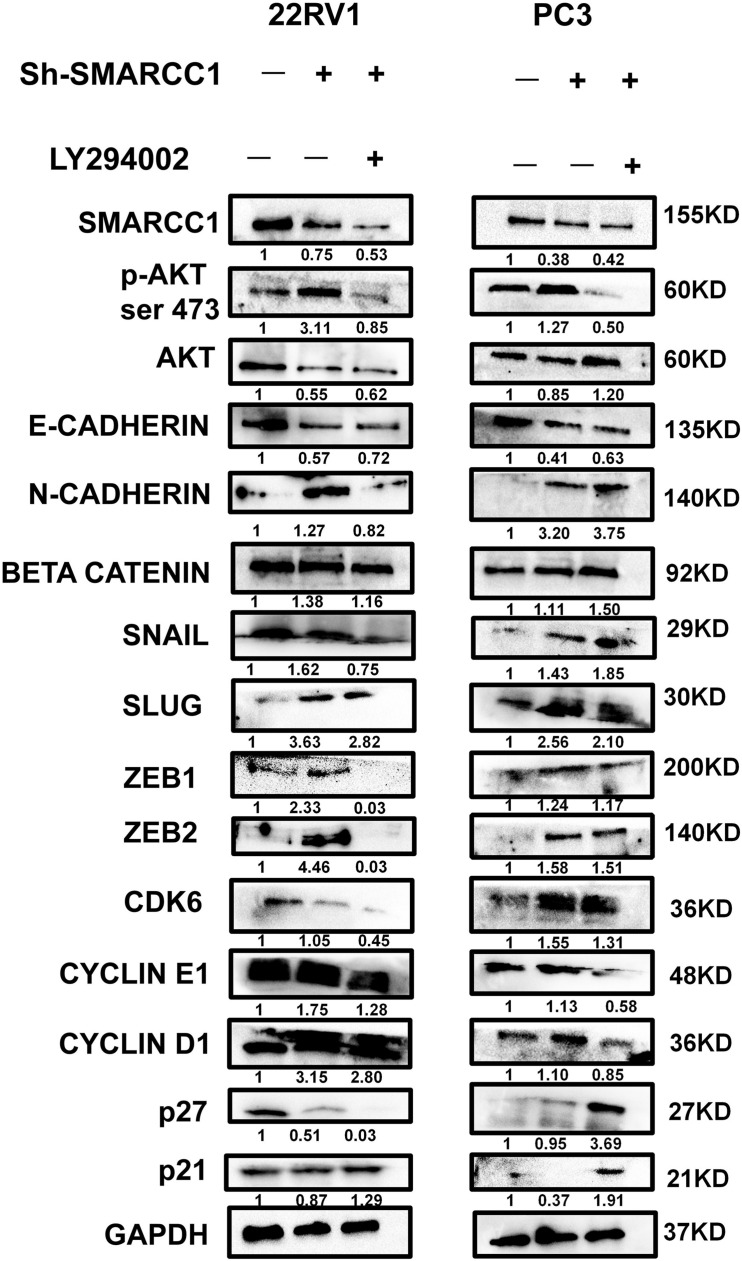
PI3K/AKT pathway blockade with LY294002 partially reversed the protein expression in *SMARCC1* knockdown PCa cells. The gray density values are indicated under the corresponding bands.

*SMARCC1* knockdown activated the *PI3K/AKT* pathway in PCa cells, as indicated by the elevated phosphorylation of *Akt* at ser-473 and thr-308 (Figures 5A,B). Activation of the *PI3K/AKT* pathway stabilizes β-catenin and promotes its nuclear translocation, wherein it regulates the transcription of target genes. Loss of *SMARCC1* significantly increased the accumulation of β-catenin in the nuclear fraction of PCa cells (Figures 5C,D). Furthermore, the *PI3K/AKT* pathway blockade with the specific inhibitor LY294002 reversed the pro-proliferative and pro-metastatic effects of *SMARCC1* knockdown (Figure 6) without completely altering the expression levels of EMT and proliferation-related factors. This indicates that the *PI3K/AKT* pathway may partly mediate the pro-oncogenic effect induced by *SMARCC1* knockdown in PCa, and other pathways and mechanisms may also be involved (Figure 7).

## Discussion

High frequent mutations of several key epigenetic factors, including Rb1 and BRCA, have been revealed to induce an aggressive phenotype at the terminal stage of PCa and reflect a promoting effect of epigenetic dysregulation on PCa progression. As one of the core subunits, *SMARCC1* belongs to the SWI/SNF complex, which functions as a key epigenetic complex on genome transcription and consists of 12–14 subunits, including adenosine triphosphatase (ATPase), core, and other accessory subunits (Reisman et al., 2009; Shain and Pollack, 2013; Hohmann and Vakoc, 2014; Alver et al., 2017; Lu and Allis, 2017; Savas and Skardasi, 2018; Lei et al., 2019). A high frequency of mutations with function loss in coding genes of the SWI/SNF complex has been identified by whole genome sequencing in various cancers, especially renal carcinoma and melanoma, implying its important role in tumor suppression (Shain and Pollack, 2013; Stachowiak et al., 2020; Tsuda et al., 2021; Wang et al., 2021; Zhou et al., 2021). Mechanistically, the loss of function of the SWI/SNF complex promotes the transcription of genes related to proliferation and dedifferentiation, impairs DNA repairs, and reduces the antagonistic effect on the PRC complex (Nagl et al., 2005; Wilson et al., 2010; Tolstorukov et al., 2013; Alver et al., 2017; Stanton et al., 2017; Aras et al., 2019; Ribeiro-Silva et al., 2019; Hu et al., 2020).

However, the role of the *SWI/SNF* complex is ambiguous and controversial in PCa (Deocampo et al., 2004; Hong et al., 2005; Link et al., 2005; Heebøll et al., 2008; Hansen et al., 2011; Lee and Roberts, 2013; Prensner et al., 2013). Several studies show that it promotes PCa initiation and progression by transactivating the androgen receptor (Deocampo et al., 2004; Hong et al., 2005; Link et al., 2005), but there are also reports that some subunits function as tumor suppressors (Hansen et al., 2011; Lee and Roberts, 2013; Prensner et al., 2013). For instance, the long non-coding RNA *SChLAP1* promoted an aggressive PCa phenotype by antagonizing the *SNF5* subunit (Lee and Roberts, 2013; Prensner et al., 2013). The effect of *SMARCC1* in PCa is likewise still ambiguous and controversial. One study demonstrated the upregulation of *SMARCC1* in PCa tissues relative to benign prostate tissues (Heebøll et al., 2008), whereas a retrospective study found that the positive staining of *SMARCC1* in PCa tissues correlated with prolonged survival among local PCa patients (Hansen et al., 2011). In this study, we systematically elucidated the expression and role of *SMARCC1* in PCa. Our findings demonstrated that *SMARCC1* was significantly downregulated in PCa tissues with GS > 7, and its low expression correlated with shortened disease-free survival. In addition, silencing of *SMARCC1* in PC-3, 22RV1, and LNCaP cells significantly increased cell proliferation by promoting entry into the S phase of the cell cycle and facilitated cell migration by inducing EMT. The *in vivo* studies using a murine model showed that *SMARCC1* knocking down led to the acceleration of tumor growth and lung metastasis. These observations demonstrated the tumor-suppressive role of *SMARCC1* in PCa.

Infinite proliferation is an important hallmark of tumors (Nagl et al., 2005; Hu et al., 2020). Timing of proliferation depends on the transition speed of cell cycle and is directly associated with the expression of cell cycle-related genes (Nagl et al., 2005; Carrassa, 2013; Hu et al., 2020). Cell cycle transition through different checkpoints is driven by specific cyclins and cyclin-dependent kinases (*CDKs*) and blocked by *CKIs* that inhibit cyclins and *cyclin*–*CDK* complexes (Nagl et al., 2005; Hu et al., 2020). It has been reported that component loss of the *SWI/SNF* complex downregulates *CKIs* and upregulates cyclins at the transcriptional level (Nagl et al., 2005; Hu et al., 2020). Consistent with previous reports, we found that *SMARCC1* knockdown accelerated cell cycle transition and induced a hyper-proliferative phenotype in PCa cell lines by upregulating G1/S-specific protein *cyclin D1/E1* but downregulating cyclin-dependent kinase inhibitors *p21* and *p27*, implying that *SMARCC1* may be a potential druggable target for cell cycle checkpoint pathway in PCa.

Subunit loss in the SWI/SNF complex induces dedifferentiation phenotype, while EMT results from dedifferentiation in cancer cells (Alver et al., 2017; Wang and Unternaehrer, 2019). Previously, studies have indicated that the subunit loss of the *SWI/SNF* complex may facilitate the metastasis of tumor cells by inducing EMT in colon and gastric carcinoma (Yan et al., 2014; Wang et al., 2019). EMT is triggered by transcription factors like *Snail*, *Slug*, and *Zeb1* that repress the epithelial factor *E-cadherin* and endows the cells with greater metastatic abilities (Yan et al., 2014; Wang and Unternaehrer, 2019; Wang et al., 2019). However, it was uncertain whether the suppression of *SMARCC1* would induce EMT in PCa. We, therefore, analyzed the key proteins of EMT and found that silencing of *SMARCC1* resulted in a remarkably increased expression of vimentin and N-cadherin as well as a reduced expression of E-cadherin, which are considered as characteristic features of EMT.

It has been reported that the component loss in the *SWI/SNF* complex promotes the malignant progression of rhabdomyosarcoma and ovary carcinoma *via* activation of the *PI3K/AKT* pathway (Foster et al., 2009; Bitler et al., 2015). In PCa, activation of the PI3K/AKT pathway promotes the nuclear translocation of β-catenin, which triggers the expression of proliferation and EMT-related genes and specifically promotes tumor progression by transactivating androgen receptor and its downstream pathway (Sharma et al., 2002; Hennessy et al., 2005; Carnero, 2010; Noorolyai et al., 2019). However, the specific role of the PI3K/Akt pathway in PCa with *SMARCC1* loss still needs to be investigated. Previous studies revealed the synergistic effect of the SWI/SNF complex with other epigenetic factors, including histone acetylase and Rb1, on genome transcription (Strobeck et al., 2000; Chatterjee et al., 2018), while *SMARCC1* functions as a scaffold structure in the SWI/SNF complex to undertake coordination with other epigenetic factors (Chatterjee et al., 2018), even though we observed a significant upregulation of β-catenin in the nuclear fraction of PCa cells with *SMARCC1* depletion in our study, indicating that the *PI3K/AKT* pathway mediates the pro-oncogenic effects of *SMARCC1* loss. However, the PI3K/AKT inhibitor LY294002 only partially reversed these pro-oncogenic effects, which indicates the potential involvement of other epigenetic factors. Taken together, *SMARCC1* may function as a tumor suppressor in PCa along with other epigenetic factors, which warrant further investigation.

**FIGURE 8:**
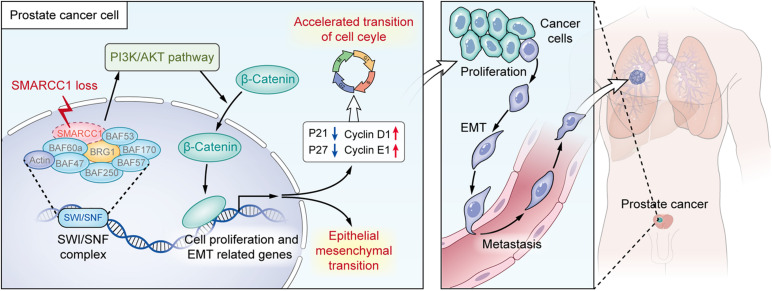
Illustration for the pro-oncogenesis effect of *SMARCC1* loss on PCa. *SMARCC1* loss in PCa activates the PI3K/AKT pathway and induces the translocation of β-catenin to promote proliferation and epithelial mesenchymal transition.

In summary, the downregulation of *SMARCC1* is correlated with a poor prognosis and an aggressive phenotype of PCa. *SMARCC1* depletion facilitates PCa cell proliferation by promoting cell cycle progression and enhanced cell migration by EMT. In addition, *SMARCC1* loss activates the PI3K/Akt signaling pathway, which plays a key role in the progression of PCa. Therefore, *SMARCC1* may be a promising therapeutic target in PCa, especially for cases with low expression levels (Figure 8).

## Data Availability Statement

The raw data supporting the conclusions of this article will be made available by the authors, without undue reservation.

## Ethics Statement

The studies involving human participants were reviewed and approved by the Ethics Committee of Nanfang Hospital, Southern Medical University. The patients/participants provided their written informed consent to participate in this study. The animal study was reviewed and approved by the Ethics Committee of Nanfang Hospital, Southern Medical University.

## Author Contributions

Z-MX and D-JL performed the material preparation, data collection, and data analysis. Y-ZY, CW, and TW assisted in collecting data. Z-MX wrote the first draft of the manuscript. D-JL revised the manuscript. S-CZ and D-JL executed the funding acquisition. S-CZ supervised the study. All authors contributed to the study conception and design, commented on previous versions of the manuscript, read, and approved the final manuscript.

## Conflict of Interest

The authors declare that the research was conducted in the absence of any commercial or financial relationships that could be construed as a potential conflict of interest.

## Footnotes

1 http://gepia.cancer-pku.cn/

## Abbreviations

PCa: prostate cancer
*SMARCC1*: SWI/SNF-related matrix-associated actin-dependent regulator of chromatin subfamily C member 1
BPH: benign prostatic hyperplasia
GS: Gleason score
SWI/SNF complex: Switch/sucrose non-fermentable complex
H&E staining: hematoxylin and eosin staining
PSB: phosphate buffer solution
siRNA: small interference RNA
shRNA: short hairpin RNA
MOI: multiplicity of infection, qRT-PCR, quantitative real-time polymerase chain reaction
cDNA: complementary DNA
GAPDH: glyceraldehyde-3 phosphate dehydrogenase
RIPA: radio-immunoprecipitation assays
PMSF: phenylmethylsulfonyl fluoride solution
SDS-PAGE: sodium dodecyl sulfate polyacrylamide gel electrophoresis
PVDF: polyvinylidene fluoride
HRP: horseradish peroxidase
DMSO: dimethyl sulfoxide
SEM: standard error of mean
CDK: cyclin-dependent kinase
CKI: cyclin-dependent kinase inhibitor
EMT: epithelial to mesenchymal transition
MMP2: matrix metalloproteinase 2
PBST: PBS-tween solution
PI3K/AKT: phosphatidylinositol 3 kinase/protein kinase B
Rb1: retinoblastoma-associated protein 1
BRCA: breast cancer susceptibility gene
ATPase: adenosine triphosphatase.
